# Keratoconus: Challenges and Emerging Trends

**DOI:** 10.4172/1747-0862.1000367

**Published:** 2018-09-30

**Authors:** D Karamichos

**Affiliations:** 1 Department of Ophthalmology, Dean McGee Eye Institute, University of Oklahoma Health Science Center, Oklahoma City, USA; 2 Department of Cell Biology, University of Oklahoma Health Sciences Center, Oklahoma City, USA

## Introduction

Keratoconus (KC) is a bilateral corneal dystrophy that affects 1:400 – 1:2000 people worldwide. It is thought to be multifactorial with the onset linked to genetic, environmental, biomechanical, and hormonal cues. Because of the multiple factors involved, KC pathophysiology remains a mystery. KC is known to manifest during puberty and develop at different, unexplained rates amongst individuals. KC affects both males and females, as well as different ethnicities with a higher prevalence towards Asian populations. There are several ocular associations reported in literature including; retinitis pigmentosa, microcornea, ectopia lentis, lenticonus, macular coloboma, and floppy eyelid syndrome. Systemic associations are also known including; Down’s syndrome, Ehlers-Danlos syndrome, and mitral valve prolapsed.

Several approaches have been deployed, *in vitro* and *in vivo* in order to determine the reason(s) that the human keratoconic cornea thins and protrudes. Genetic studies are perhaps the largest, in terms of participants/patients. Multiple genes have been proposed, including, VSX, COL1A1, COL5A1, and LOX, but studies remain inconclusive [1–13]. As a result, we have yet to see the development of an animal model that recapitulates the KC phenotype. These hurdles have hampered our progress towards unravelling the key molecular mechanisms responsible for the KC onset and progression. Perhaps the biggest gap in our knowledge about KC is that very few studies have attempted to link *in vitro* findings to those *in vivo*.

## Sex hormones

Sex hormones are known to play a role in the maintenance of the structure and integrity of the human cornea. Hormone levels have been reported to alter corneal thickness, curvature, and sensitivity during different times of the menstrual cycle and pregnancy. Furthermore, the presence of hormones in the human tear film has also been reported. Surprisingly, the role of sex hormones in corneal diseases has not been investigated. Our group recently suggested that KC is at least partially a systemic disease. We reported hormone alterations in the KC-derived corneal stromal cells as well as in human KC saliva [14]. Since then, our work is spearheading the field with what we believe is the discovery of the first-ever KC biomarker. If we are correct about our hypothesis, and our gene candidate is truly a KC biomarker, it could transcend the landscape of KC research, KC treatment, and KC care.

## Prolactin-induced protein

Prolactin-Induced Protein (PIP) is also known as gross cystic disease fluid protein 15 (GCDFP-15), extra-parotid glycoprotein (EPGP), and gp17 seminal actin-binding protein (SABP) [15–17]. PIP is tightly linked to sex hormones and is the connection that initiated our hypothesis that PIP may be involved in KC pathogenesis. PIP is upregulated by androgens and downregulated by estrogens, although the exact downstream signaling pathway(s) is not yet well defined.

PIP was originally discovered as a marker for both benign and malignant apocrine metaplasia, considering that the protein is not normally expressed in healthy breast tissue [18]. In KC, we were the first to report PIP modulation, showing significant downregulation in both KC-derived corneal stromal cells and KC tear fluids, when compared to healthy controls [19]. This was intriguing, especially in regard to the interplay between PIP and sex hormones, and the effects of hormonal imbalances in the human cornea (ex. corneal thinning during pregnancy). Amazingly, the role of sex hormones in KC had never been investigated in depth, prior to our studies. Our recent comprehensive review on PIP and KC highlights this emerging topic [20].

## Future Implications

Our recent findings spearhead the work done in the KC field with regard to biomarker discovery and validation. Defining a universally agreed upon path for biomarker validation is urgently needed to circumvent many of the hurdles faced in KC prognosis.

If PIP is confirmed as a biomarker, a clinical test/exam could be quickly implemented for any individual that is suspected to have KC. Clinical implications would be significant, since KC could be diagnosed earlier, and clinicians can develop a more comprehensive plan of treatment. The test/exam could also be combined with current imaging techniques, such as Pentacam^®^. Pentacam^®^ is currently the gold standard in anterior segment tomography and one of the most important examinations for KC suspects. In the future, PIP-focused treatments could be developed for the benefit of KC patients. Overall, a discovery such as PIP would be a breakthrough and may lead to better prevention and management of the disease.
